# Does worriedness among the rural adults promote COVID-19 related awareness in Bangladesh?

**DOI:** 10.1016/j.heliyon.2021.e06556

**Published:** 2021-03-19

**Authors:** Muhammad Mahmudul Hasan, Ashis Talukder, Muhammad Khairul Alam, Muhammad Kausar Hossain, Asikunnaby

**Affiliations:** a Department of Statistics, University of Dhaka, Bangladesh; b Department of Mathematical Sciences, Durham University, UK; c Statistics Discipline, Khulna University, Bangladesh; d Department of Statistics, Jahangirnagar University, Bangladesh; e Department of Finance, University of Dhaka, Bangladesh; f Department of Disaster and Human Security Management, Bangladesh University of Professionals, Bangladesh; g Institute of Hazard, Risk, and Resilience, Durham University, UK

**Keywords:** Worriedness, Rural adults, Awareness, Binary logistic regression, Social distancing

## Abstract

People living in urban areas are usually more aware of their health issues due to the availability and accessibility of health care facilities. Several studies have illustrated anxiousness, attitudes, and perceptions among urban people during COVID-19. This research attempted to assess how worriedness among rural adults may promote COVID-19 related awareness in Bangladesh. A cross-sectional online survey of 311 respondents aged 18 or greater was conducted through Facebook focusing only on the people living in rural areas. The survey included a consent form and requested demographic as well as pandemic related information in a three-section questionnaire from the respondents. We used the chi-square test statistic for bivariate analysis and the binary logistic regression model along with some tools to validate the model to analyze the impact of worriedness on awareness. The bivariate result showed a significant association among regular hand washing (p=.007), knowledge about the proper amount of time for washing one's hands effectively (p=.004), rules of social distancing (p=.00), and education level (p=.046) with our outcome variable worriedness. From our binary logistic regression model fitting, it emerged that the females (p=.032, OR=.729) who regularly wash their hands (0R=.393, p=.023), know the rules of social distancing for “yes” (0R=14.525,p< .01), and “no” groups (0R= 5.518, p< .01), and age groups (18–27, 28 to 37, 38 to 47) were more worried. Results from our modeling justify an accuracy of 73.08%, a sensitivity of 93.71%, and a specificity of 29.33% with Cohen's kappa statistic = .2716, suggesting a fair model fitting. This study shows that the current COVID-19 situation created awareness among females and adults aged between 18 to 47 years in rural Bangladesh.

## 1 Introduction

Coronaviruses are a family of RNA viruses that cause diseases in mammals and birds. Coronaviruses are considered responsible for the Middle East Respiratory Syndrome (MERS), the Severer Acute Respiratory Syndrome (SARS), and for Coronavirus Disease 2019 (COVID-19) (Branswell, 2015). COVID-19 was first diagnosed in China on 31 December 2019 (WHO, 2020a), this deadly virus spread rapidly all over the world and is still affecting people. On 11 March 2020 the World Health Organization (WHO) declared COVID-19 as a pandemic due to its rapid spread over 114 countries with 4,291 deaths (WHO, 2020a). As of 2 March 2021, the global number of cases has gone beyond 11.5 million with 2.55 million deaths approximately (Worldometers, 2021).

The best preventative measure to reduce the spread of coronavirus is to obey the rules advised by the World Health Organization and the respective government officials (WHO, 2020b). A vaccine for the coronavirus disease and being aware of one's safety as well as others should be an immediate priority. The coronavirus mainly spreads by the droplets of infected persons, so people should maintain some precautions to avoid contracting the virus such as: washing hands regularly, maintaining social distancing (at least 2 m/6 feet), avoiding unnecessary outings, and so on (WHO, 2020c).

Several studies have analyzed how social awareness can impact the spread of coronavirus throughout the world. A study conducted in India through an online survey revealed that respondents who had a median level of knowledge about the coronavirus were eager to follow central government instructions, and 72% considered hand sanitizer as an important preventative factor (Deblina et al., 2020). In 2015, a study was conducted in Saudi Arabia to assess the knowledge and attitudes of over 1,147 adults which found that for both genders, knowledge was a significant factor for the consciousness about the disease (Almutairi et al., 2015), which was regarding to MERS epidemic. Another questionnaire-based online survey was conducted with 210 people during COVID-19, it suggested that social distancing heavily depends on awareness and it is important (p<0.00) at a 1% level of significance (Qazi et al., 2020). Moreover, authors from a study conducted during the MERS-CoV outbreak found that there were differences among Saudi Arabian people and other Arabic speaking countries concerning the extent of participants’ awareness about the virus (Althobaity et al., 2017). Another study in China with 507 patients reported between 13 January 2020 to 31 January 2020 recommended that news reports and virtual media are important factors in reducing the spread of the coronavirus (Sun et al., 2020). Thus, awareness is likely a critical factor to examine in the current pandemic climate for Bangladesh.

In Bangladesh, the first case of COVID-19 was detected on 8 March 2020 and the lockdown came into effect on 26 March 2020 (Directorate General of Health Services [DGHS], 2020). For a highly dense country like Bangladesh, it is very difficult to maintain social distancing especially when only 15% (making $30 > per week) of the country's total population can afford not to work (Faisal, 2020). A study was conducted by the Bangladesh Rural Advancement Committee (BRAC) with 2,317 people and almost 95% of people are on their way to losing income due to the spread of COVID-19 in Bangladesh (BRAC, 2020). As of 2 March 2021, the number of cases has reached 546,801 with 8,416 deaths; however, people are not aware despite the increasing number of cases (Worldometers, 2021). In a study on Bangladesh, respondents suggested that the government is taking necessary steps to increase its people's awareness (Mumu et al., 2020). Although the Bangladeshi government took strict measures in the urban areas, the new concern is for those living in rural areas. Different studies (Anwar, 2020; Javed, 2020; Mumu et al., 2020) were conducted on those living in the urban areas of Bangladesh, and those studies recommended necessary steps to raise consciousness among mass people. This research is an attempt to explore the worriedness, perception, awareness, and knowledge on the health issues among rural adults of Bangladesh during the ongoing pandemic situation and contribute towards the existing knowledge gap on the same issue.

## 2 Material and method

### 2.1 Data and variables

This study was conducted with an online-based questionnaire survey using Google Forms. The questionnaire was designed into 3 sections: consent section, demographic information, and questions on the current pandemic in the last section. The respondents were adults who use Facebook and understood questions in English, we sent the link through Facebook Messenger only to those living in rural areas. The Facebook policy for opening an account is 13 years of age; as our focus was on rural adults, only respondents aged 18 or older were considered for this study. The data were collected from 15 May 2020 to 2 June 2020. Only 318 respondents participated in our study, this may be because rural adults may not have as much access to internet facilities as the urban adults. Out of those 318 respondents, only 7 respondents did not agree to give consent and we discarded those as missing values.

We collected data on age, education level, religion, and gender as our demographic variables. The variable age was classified into six different categories (18–27, 28 to 37, 38 to 47, 48 to 57, 58 to 67, and 68+); education level was classified with a 5-point Likert scale and categorized as no education, primary education (class 1 to 5), secondary education (class 6 to 10), higher secondary education (class 11 and 12) and tertiary education (university level); the religion variable was divided into 3 categories (Hindu, Muslim, and Others), and gender had 2 categories (male, female).

For our pandemic related questions, we asked how they first heard about the pandemic (social media, friends and family, and news media), if they wash hands regularly (yes or no), their knowledge about the proper time length for washing one's hand effectively (yes or no), how they perceived the coronavirus (naturally occurred or man-made), if they go out regularly (yes or no), do they know the rules of social distancing (yes, no, or maybe), and if they had worriedness about the pandemic situation (yes or no). The variable worriedness is our response variable; we have considered other demographic and pandemic variables as our covariates. The variables related to going outside, knowing the rules of social distancing were treated as awareness in our study.

### 2.2 Statistical analysis

The association among the covariates of our study and worriedness were examined both in bivariate and multivariate frameworks. In a bivariate framework, we used the chi-square test (Pearson, 1893) of independence to assess the associations between worriedness with other covariates.

The expression for chi-square is,

(1) $$\chi^{2}=\overset{n}{\underset{j=1}{\sum }}\frac{{\left(A_{j}-B_{j}\right)}^{2}}{B_{j}}$$

Where $A_{j}$ is the observed and $B_{j}$ is the expected cell frequencies. The test statistic follows a chi-square distribution with (m−1)(n−1) degrees of freedom, where m is the number of categories of the covariates and n is our response variable.

In the multivariate layout, we used the binary logistic regression model (Freedman, 2009), and it addressed the classification problem using the maximum likelihood estimation procedure to determine the parameters of interest for this study. Let $A_{i}\left(i=1,2,\ldots ,n\right)$ be our outcome variable with categories 1,...,j,…,c and $b_{i}={\left(b_{i1},b_{i2},\ldots ,b_{in}\right)}^{\prime }$ as the column vector of k covariate values. The form of our logistic regression model can be expressed mathematically as,

(2) $$log\left[\frac{\nabla }{1-\nabla }\right]=\alpha_{j}+\beta^{\prime }b_{i}\;For\;j=1,2,\ldots ,c-1$$

Where ∇ represents the probability of occurrence, and 1−∇ is the probability of the event of interest not happening.

### 2.3 Model evaluation

To evaluate the model, we have considered some criteria, such as accuracy checking, sensitivity analysis, specificity, and Cohen's kappa (Khemphila et al., 2010).

**Accuracy:** It is the main basis for analyzing the predictive measure of modeling. Mathematically the accuracy can be estimated as follows,

$$Accuracy=\frac{True\;Positive+True\;Negative}{True\;Positive+False\;Negative+True\;Negative+False\;Positive}$$

**Sensitivity:** The percentage of the real positive cases which are predicted as positive can be expressed as follows,

$$Sensitivity=\frac{True\;Positive}{True\;Positive+False\;Negative}$$

**Specificity:** The percentage of the real negative cases which are predicted as positive can be expressed as follows,

$$Specificity=\frac{True\;Negative}{True\;Negative+False\;Positive}$$

**Cohen's Kappa:** It analyzes the percentage between the predicted and the actual classifications in a dataset. The value of Cohen's kappa statistic is less than or equal to 1. The measurement can be categorized as 0 is treated as “no agreement,” .01 to .20 considered as “slight,” .21 to .40 as “fair,” .41 to .60 treated as “moderate,” .61 to .80 as “substantial,” and .81 to 1 as an “almost a perfect agreement” (McHugh, 2012).

### 2.4 Ethical statement

Consent was taken online before going through section 2 of our survey. Participants' anonymity and confidentiality of data were ensured, then participants were provided with information about the nature, purpose, and procedure of this study. The participants had the right to withdraw their data from the study. The study protocol was approved by the Ethical Clearance Committee of Khulna University. Although this research is not related to human trials, our research has been conducted following the Declaration of Helsinki ethical standard.

## 3 Results

### 3.1 Bivariate analysis

The outputs of our bivariate analysis are presented in Table 1. The results of our analysis (from Table 1) show a significant association among regular hand washing (p=.007), knowledge about the proper time length of hand washing (p=.004), social distancing (p<0.00), and education level (p=.046) with our outcome variable worriedness; the results are statistically significant (*p* < .05). From our chi-square test estimation, the variables of age (p=.175), gender(p=.252), religion (p=.807), first knowledge about COVID-19 (p=.257), perception(p=.697), and going outside (p=.317) showed insignificant associations with worriedness. Out of 311 respondents, 204 washed their hands regularly, 196 knew the proper time length of hand washing, and 197 knew the rules of social distancing. Most of our participants were between 18 and 27 (n = 153) likely because younger people in the rural areas are more frequent users of Facebook; out of these 153, 68% were worried about the pandemic. In addition, 70% of the Hindus, 67.4% of the Muslims, and 80% of respondents from other religions were also worried about the pandemic situation, though, these results are statistically insignificant. We also found that females are primarily worried about the pandemic. Most of the respondents first heard about coronavirus from friends and family members. Also, most of the respondents think of coronavirus as naturally occurring than the virus being man-made.

**Table 1:** Assessing the association between covariates and worriedness using the chi-square test(n=311).

Covariates	Worriedness about the COVID-19 pandemic		Chi-square (Degrees of Freedom)	p-value
	Yes	No		
**Age (in years)**				
18–27	104 (68.0%)	49 (32.0%)		
28–37	84 (73.7%)	30 (26.3%)		
38–47	9 (50.0%)	9 (50.0%)	7.671 (5)	.175
48–57	3 (42.9%)	4 (57.1%)		
58–67	7 (63.6%)	4 (36.4%)		
68+	4 (50.0%)	4 (50.0%)		
**Religion**				
Hindu	21 (70.0%)	(30.0%)		
Muslim	186 (67.4%)	90 (32.6%)	.429 (2)	.807
Others	4 (80.0%)	1 (20.0%)		
**Gender**				
Male	112 (65.1%)	60 (34.9%)	1.314 (1)	.252
Female	99 (71.2%)	40 (28.8%)		
**First knew about COVID-19 through**				
Facebook	59 (70.2%)	25 (29.8%)		
Friends and Family	103 (70.5%)	43 (29.5%)	2.716 (2)	.257
News	49 (60.5%)	32 (39.5%)		
**Washing hands regularly**				
Yes	204 (69.6%)	89 (30.4%)	7.344 (1)	.007
No	7 (38.9%)	11 (61.1%)		
**Knowledge about the proper time length of washing hands**				
Yes	196 (70.5%)	82 (29.5%)	8.484 (1)	.004
No	15 (45.5%)	18 (54.5%)		
**Perception**				
Naturally occurred	121 (68.8%)	55 (31.2%)	.152 (1)	.697
Man-made	90 (67.8%)	45 (32.2%)		
**Going out regularly**				
Yes	66 (64.1%)	37 (35.9%)	1.002 (1)	.317
No	145 (69.7%)	63 (30.3%)		
**Knows social distancing rules**				
Yes	197 (74.6%)	67 (25.4%)		
No	4 (22.2%)	14 (77.8%)	37.527 (2)	.000
Maybe	10 (34.5%)	19 (65.5%)		
**Education Level**				
No education	67 (75.3%)	22 (24.7%)		
Primary	52 (72.2%)	20 (27.8%)		
Secondary	43 (62.3%)	26 (37.7%)	9.681 (4)	.046
Higher Secondary	24 (72.7%)	9 (27.3%)		
Tertiary	25 (52.1%)	23 (47.9%)		

### 3.2 Regression analysis

The main intent of this study was to assess the link between worriedness with other factors in rural Bangladesh. To identify the factors associated with worriedness a binary logistic regression model was considered and the results are presented in Table 2. It can be said that the variable gender has a significant (*p* = .032) effect on our outcome variable worriedness. The odds of worriedness for the male gender is [(.729−1) × 100] = 27% lower compared to the female gender (0R=.729,p<.05). Besides this covariate, the odds of worriedness for a regular hand washer is approximately 61% lower compared to irregular hand washers (0R=0.393,p<0.05), the odds of worriedness on the knowledge about rules of social distancing is higher for both the yes and no group respondents compared to the maybe respondents (0R=14.525,p<.01), and (0R=5.518, p<.01) respectively. The age groups (18–27, 28 to 37, and 38 to 47) are more worried compared to the other ages of rural people in Bangladesh. These results are statistically significant at the 5% level of significance. The variables' first knowledge about COVID-19, perception, knowledge about the proper time length of hand washing, going out regularly, and education levels revealed insignificant results.

**Table 2:** Odds ratios with p-values of associated factors for worriedness obtained from the logistic regression model (OR = odds ratio; CI = Confidence Interval).

Factors	Estimate	OR [95% CI]	p-value
**Intercept**	-1.995	.136	.009
Gender (ref: Female)			
Male	-.316	.729 [.546, .973]	.032
**First knew about COVID-19 (ref: News media)**			
Facebook	-.043	.958 [.690, 1.331]	.798
Friends and family	.075	1.078 [.724, 1.605]	.712
**Washing hands regularly (ref: No)**			
Yes	-.934	.393 [.176, .879]	.023
**Knowledge about the proper time length of washing hands (ref: No)**			
Yes	.553	1.738 [.962, 3.141]	.067
**Going out regularly (ref: No)**			
Yes	.151	1.163 [.856, 1.581]	.333
**Knows social distancing rules (ref: Maybe)**			
Yes	2.676	14.525 [4.439, 47.530]	.000
No	1.708	5.518 [2.842, 10.711]	.000
**Education Level (ref: Tertiary)**			
No education	.034	1.035 [.724, 1.480]	.851
Primary	.489	1.630 [1.093, 2.432]	.017
Secondary	-.237	.789 [.481, 1.295)]	.348
Higher Secondary	.690	1.993 [1.244, 3.194]	.004
**Age (in years) (ref: 68+)**			
18–27	1.596	4.933 [1.457, 16.700)]	.010
28–37	1.457	4.291 [1.258, 14.633]	.020
38–47	2.362	10.613 [2.716, 41.475]	.001
48–57	.710	2.035 [.391, 10.580]	.398
58–67	.854	2.349 [.580, 9.509]	.231

### 3.3 Diagnostic checking

The prediction results with performance parameters are displayed in Table 3. Utilizing the Logistic regression model, the accuracy in the dataset is seen as 73.08% with a sensitivity of 93.71% and specificity of 29.33%. The estimation of Cohen's kappa statistic for our data set is .2716 recommending a “fair” discriminative power.

**Table 3:** The performance indicators of the model fitting.

Indicators	Value
Accuracy	.7308
95% Confidence Interval	[.6691, .7865]
Cohen's Kappa	.2716
Sensitivity	.9371
Specificity	.2933

**Figure 1:**
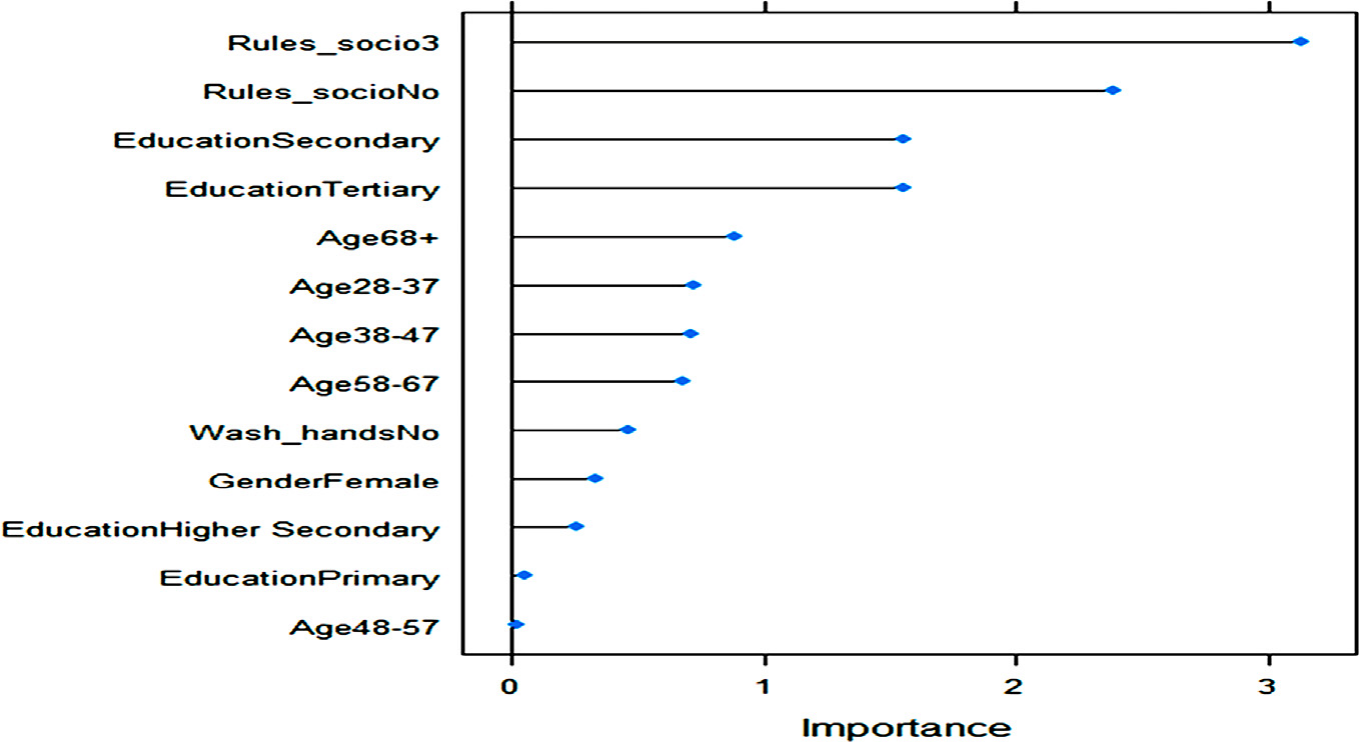
Important categories responsible for causing worriedness among respondents.

We also identified the most important categories that were responsible for causing worriedness among survey respondents in Figure 1. According to the results, maintaining the rules of social distance (maybe) for the last group are most significant, followed by maintaining the rules of social distancing of the no group, the secondary and tertiary education levels, the age groups: 68+, 28 to 37, 38 to 47, and 58 to 67, the washing hands regularly no group, female gender, higher secondary and primary education levels, and the age group of 48–57.

## 4 Discussion

The focus of our research work was to find the factors associated with worriedness among the rural people of Bangladesh. To fulfill our objective, we first explored the chi-square test of independence to assess the associations among the variables with worriedness and then used the binary logistic regression model with model evaluation using the performance measures accuracy, specificity, sensitivity, and Cohen's Kappa measurement.

We collected 311 respondents’ information who live in rural areas of Bangladesh, 172 respondents out of 311 were male and most of them belong to the 18 to 27 age group.

For our bivariate analysis, we used the chi-square test statistic to assess the association among the variables with our response variable worriedness and found the variables regular hand washing, knowing the rules of social distancing, knowledge about the duration of a proper hand wash, and education level as significant. We have compared the results from this study with the existing works of literature, and our research found similar conclusions as previous research (Deblina et al., 2020) involving the knowledge about social distancing as a significant factor. A recent cross-sectional (Tripathi et al., 2020) was conducted in the South-west of Saudi Arabia to assess the awareness among social workers and the study emerged social distancing and hand hygiene as significant factors for awareness like this research. So, a previous study as well as this study strongly suggested that washing the hands regularly and maintaining social distancing are the most significant factors for reducing the spread of coronavirus. We also compared this study with another that was conducted in a rural area of Saudi Arabia (Tripathi et al., 2020) and our results are in line with those reported for the rural population in Saudi Arabia.

Although we considered several protective health behaviors, self-efficacy regarding the protective health actions recommended by the government is a key for controlling the outbreak of the virus (Roma, 2020). A previous study (Roma, 2020) highlights a relationship between self-efficacy and compliance with the protective measures during this outbreak. Our binary logistic regression model revealed significant results for gender, certain age groups (18–27, 28 to 37, and 38 to 47), regular hand washing, and knowledge about social distancing rules. A recent study found that young age groups were associated with higher distress, which deviates from our results (Roma, 2020). According to our findings, older people tended to report higher worriedness; therefore, in an effort reduce such feelings they should comply with the proposed measure to reduce COVID-19 exposure, particularly those associated with hygiene (Nivette et al., 2020).

The accuracy of our fitted model is 73.08% with a sensitivity of 93.71%, which can be considered as a fair model based on Cohen's Kappa measurement. We have shown graphically that important factors for creating the awareness among rural adults in Bangladesh by ascending order of their importance. From the figure, rules of social distancing and education level emerged as the most significant factor.

This study has a few limitations. We have only considered individuals who have internet access and use Facebook. We have also kept our questionnaire short so that respondents can answer even with poor internet access from the rural areas of Bangladesh. Working on these limitations would help to improve the predictive accuracy versus having to pick up the calculations on abstract judgment. However, our current research work is a simple attempt to analyze the real scenario of rural people living in Bangladesh.

We would strongly recommend conducting a broad survey to find out the overall scenario of Bangladesh. The government needs to take necessary steps to increase awareness among the rural people by providing easy internet access. The news media could broadcast frequent contents on social distancing, rules of proper hand washing, and educated people who live in rural areas could also play a vital role to increase consciousness among the rural laypeople of Bangladesh.

## 5 Conclusion

In this study, we have used binary logistic regression to assess the impact of worriedness with the factors related to the current COVID-19 situation. We considered variables that may relate to worriedness that could virtually identify the awareness among the Bangladeshi people. Our model fitting suggested a fair accuracy of modeling and some significant factors that create worriedness among rural people were identified. The results from this research could be useful to policymakers, as they could make more adaptive changes based on empirical evidence to look after the rural people of Bangladesh during and after the pandemic.

## Declarations

### Author contribution statement

Muhammad Mahmudul Hasan: Conceived and designed the experiments; Performed the experiments; Analyzed and interpreted the data; Contributed reagents, materials, analysis tools or data; Wrote the paper.

Ashis Talukder: Conceived and designed the experiments; Performed the experiments; Analyzed and interpreted the data; Contributed reagents, materials, analysis tools or data.

Muhammad Khairul Alam: Performed the experiments; Analyzed and interpreted the data.

Muhammad Kausar Hossain: Conceived and designed the experiments; Contributed reagents, materials, analysis tools or data.

Asikunnaby: Contributed reagents, materials, analysis tools or data; Wrote the paper.

### Funding statement

This research did not receive any specific grant from funding agencies in the public, commercial, or not-for-profit sectors.

### Data availability statement

Data included in supplementary material.

### Declaration of interests statement

The authors declare no conflict of interest.

### Additional information

No additional information is available for this paper.

## Appendix A Supplementary data

covid_data covid_data
